# Food choices characterized by the Nutri-Score nutrient profile and risk of cardiovascular diseases

**DOI:** 10.1093/eurpub/ckac129.171

**Published:** 2022-10-25

**Authors:** M Deschasaux-Tanguy, I Huybrechts, C Julia, S Hercberg, B Srour, J Danesh, E Riboli, MJ Gunter, M Touvier

**Affiliations:** 1 Nutritional Epidemiology Research Team, Inserm U1153, Inrae U1125, Cnam, Sorbonne Paris Nord University, Bobigny, France; 2 Nutrition and Metabolism Section, International Agency for Research on Cancer, Lyon, France; 3 Public Health Department, Avicenne Hospital, Bobigny, France; 4 Department of Public Health and Primary Care, University of Cambridge, Cambridge, UK; 5 Faculty of Medicine, School of Public Health, Imperial College London, London, UK

## Abstract

**Background:**

Nutrition is a well-established risk factor for cardiovascular diseases (CVD) that can be leveraged by public health prevention strategies. In addition to dietary guidelines, front-of-pack nutrition labels (FoPNL) can help consumers make healthier food choices. Nutri-Score, a scientifically validated 5-color FopNL based on the nutrient profile FSAm-NPS has been adopted by several European countries but remains optional under current EU labeling regulation, which is to be revised end of 2022. Scientific evidence is therefore needed on the relevance of the Nutri-Score at the European level. Our objective was to study the association between the consumption of food as graded by the FSAm-NPS and CVD risk in a large European population.

**Methods:**

This prospective analysis was conducted on a case-cohort comprising 13,308 participants without CVD risk factors at baseline, among which 5,326 first incident cases of CVD from the EPIC-CVD study (8 European countries). Food intakes were assessed using country-specific dietary questionnaires. The FSAm-NPS was calculated for each food based on its 100g content in energy, sugar, saturated fatty acid, sodium, fibre, protein, and fruits/vegetables/legumes/nuts. Multi-adjusted Cox models were computed.

**Results:**

Overall, associations were observed between the consumption of foods with a higher FSAm-NPS score (lower nutritional value) and a higher risk of myocardial infarction (MI; HR1-SD=1.12 [1.05,1.21]; HRQ5/Q1=1.23 [1.00,1.52]). Associations with stroke were not significant. Overall, associations were more particularly observed in men.

**Conclusions:**

In this large European population, a higher risk of MI was observed in individuals consuming on average a diet with higher FSAm-NPS foods (reflecting consumption of foods with a lower nutritional value/less favourable Nutri-Score). This adds to the evidence on the relevance of Nutri-Score as a public health tool to help consumers choose healthier food products.

**Key messages:**

• The consumption of foods with a lower nutritional quality as graded by the Nutri-Score was associated with a higher risk of myocardial infarction in the large European EPIC-CVD case-cohort study.

• This adds to the evidence supporting the relevance of the Nutri-Score as a complementary tool to dietary guidelines to help consumers make healthier food choices.

